# Acoustic characterization of shell and size-engineered submicron contrast agents using attenuation measurements

**DOI:** 10.1016/j.ultsonch.2026.107884

**Published:** 2026-05-15

**Authors:** Mahshid Yaali, Agata A. Exner, Michael C. Kolios

**Affiliations:** a Toronto Metropolitan University, 350 Victoria Street, Toronto, Ontario, Canada; b Institute for Biomedical Engineering, Science and Technology (iBEST), a partnership between St. Michaels Hospital and Toronto Metropolitan University, 209 Victoria St, Toronto, Ontario, Canada; c Department of Radiology, Case Western Reserve University, Cleveland, OH 44106, USA; d Department of Biomedical Engineering, Case School of Engineering, Case Western Reserve University, Cleveland, OH 44106, USA

**Keywords:** Nanobubbles, Stable cavitation, Ultrasound attenuation, Bubble dynamics, Shell stiffness, Shell friction

## Abstract

Nanobubbles (NBs), consisting of a lipid shell surrounding a gas core, have gained significant interest as contrast agents for ultrasound molecular imaging. Their acoustic response is strongly influenced by size and shell properties, yet most prior work has focused on microbubble characterization. Building on insights from microbubble studies, this work investigates the viscoelastic properties of in-house synthesized phospholipid-coated submicron NBs (average diameters of 650–720 nm) using ultrasound bulk attenuation measurements. Three NB formulations with distinct shell compositions were examined. The results highlight the critical role of shell properties in determining NB resonance frequencies. Furthermore, pressure-dependent shifts in resonance revealed strong nonlinear behavior at higher acoustic driving pressures (up to 280 kPa). Comparison with microbubbles of identical shell types showed that shell stiffness and friction are size-dependent, likely due to shell properties and the shear-thinning behavior of phospholipids. These findings provide new insights into NB dynamics with potential implications for both diagnostic and therapeutic ultrasound applications.

## 1 Introduction

Microbubbles (MBs) and submicron nanobubbles (NBs) are widely studied ultrasound contrast agents (UCAs), typically composed of a gas core (e.g., perfluoropropane, C_3_F_8_, or sulfur hexafluoride, SF_6_) encapsulated by a stabilizing shell of proteins, lipids, or polymers [1]. Lipid-coated bubbles, one of the most common UCAs, are FDA approved and broadly applied in clinical practice due to their simple fabrication and strong echogenicity. They are routinely used for ultrasound contrast enhancement [2], [3], [4], molecular imaging [5], [6], targeted drug and gene delivery [7], [8], [9], and tumor ablation [10], [11].

MBs, with diameters typically ranging from 1–8 µm, remain largely confined to the vasculature, which restricts their capacity to target biomarkers located beyond the endothelial barrier. This limits their utility for extravascular molecular targeting. In contrast, NBs, by virtue of their submicron size, can penetrate leaky vasculature and accumulate in tissues [3], [10]. This capability, along with their higher number density compared to MBs, is particularly advantageous in diseases such as cancer and type 1 diabetes, where hyperpermeable vasculature facilitates NB extravasation into the tumor microenvironment, enabling more effective biomarker targeting and tumor delineation [12], [13].

Optimizing the acoustic response of bubbles is essential for specific clinical and preclinical applications. The elastic shell plays a critical role in governing bubble dynamics. Two key viscoelastic parameters, defined as stiffness $\left(S_{p}\right)$, and friction $\left(S_{f}\right)$, are measures to show the resistance of the lipid layer to deformation during the oscillations, which changes for larger ($R>1.1R_{0}$) radial oscillations (where R_0_ is the equilibrium radius), especially during shell buckling. These parameters serve as accurate representations of the shell’s mechanical behavior. Numerous studies have modeled shell behavior and characterized MB shells using both individual bubble and bulk population approaches.

Individual bubble studies include indirect techniques, such as ultrasound stimulation, high speed microscopy, light scattering, microbubble spectroscopy, and photothermal stimulation, which have been used to extract radial responses and oscillation dynamics (Morgan et al. [14], Chetty et al. [15], Tu et al. [16], Van der Meer et al. [17], Helfield and Goertz [18], Renaud et al. [19], Lum et al. [20]). Direct methods such as Atomic Force Microscopy (AFM) [21], [22], electron microscopy [23], AFM based force spectroscopy [24], [25], [26], and Fluorescence Lifetime Imaging Microscopy (FLIM) [27] have been employed to directly probe shell stiffness, friction, and elasticity at the single-bubble level. Nawijn et al. [28] extended single-bubble characterization by applying a stress–strain framework to acoustically driven MBs, enabling the separation of elastic and viscous shell contributions without relying on boundary-constrained conditions. While these approaches provide detailed insights, they may not fully capture the dynamics under ultrasound exposure, particularly because individual bubble studies often overlook the influence of bubble concentration on collective in vivo behaviour, and AFM methods probe stiffness at different timescales.

Bulk population studies complement single-bubble approaches by investigating large ensembles of MBs. Using ultrasound attenuation, scattering, and resonance analysis, studies by Segers et al. [29], Xia and Porter [30], Goertz et al. [31], Gong et al. [32], Helfield et al. [33], Sojahrood et al. [34], and Rostam Shirazi [35] have reported on nonlinear elasticity, frequency-dependent attenuation, pressure-dependent resonance shifts, and shell viscoelastic properties across different MB formulations and sizes. More recently, Zhang et al. and Lu et al. [36], [37] used pressure-dependent attenuation measurements to track how shell mechanical properties of monodisperse MBs vary across compression regimes, revealing that shell composition (including PEGylated lipid content) governs both the mechanical response and the onset of structural instability. These studies bridge the gap between detailed laboratory measurements and clinically relevant behavior.

However, all of these investigations have been limited to MBs. Despite the increasing interest in NBs, no comparable efforts have been made to determine their shell viscoelastic parameters. In this work, we address this gap by combining bulk ultrasound attenuation measurements with a linear theoretical model to estimate the viscoelastic parameters of lipid-coated NBs with three distinct shell compositions. To the best of our knowledge, this is the first study to characterize shell stiffness and friction of submicron bubbles (mean diameters 650–720 nm) using this approach.

## 2 Methods and materials

### 2.1 Coated bubble model

The radial oscillations of lipid-coated NBs were modeled using the conventional linearized Rayleigh–Plesset equation as described in [38]. When investigating bubble dynamics, it is important to distinguish between two types of nonlinear oscillations: (i) the intrinsic nonlinear response of the bubble, where shell properties remain linear, and (ii) the additional nonlinearity introduced by lipid-coated shells, where the shell itself exhibits nonlinear behavior that depends on the incident pressure. The latter is particularly notable, as it gives rise to pronounced harmonic generation, even under very low acoustic pressures (on the order of a few kilopascals) [39], [40].

In the linear framework, the resonance frequency is the frequency at which bubbles exhibit maximum attenuation of acoustic energy. This framework is valid under the assumption of small-amplitude radial oscillations, i.e., when the driving acoustic pressure is sufficiently low that the bubble wall displacement remains a small fraction of the equilibrium radius. Under these conditions, the bubble dynamics can be linearized, and the total damping is expressed as the sum of three contributions: viscous losses in the shell (δ_sh_), viscous losses in the surrounding liquid (δ_liq_), and acoustic radiation losses (δ_rad_). This standard formulation is comprehensively treated in [38] and widely adopted in the microbubble literature [41], [42], [43]. The linearized Rayleigh–Plesset model relies on several key physical assumptions: (i) the bubble undergoes small-amplitude radial oscillations, such that the instantaneous radius R remains close to its equilibrium value R_0_ (i.e., |R − R_0_|/R_0_ ≪ 1), allowing higher-order terms to be neglected; (ii) the bubble retains perfect spherical symmetry throughout its oscillation; (iii) the surrounding liquid is treated as incompressible and Newtonian; (iv) the gas inside the bubble follows a polytropic process, characterized by the coefficient κ; and (v) the lipid shell is modeled as a thin, uniform viscoelastic layer. Under these assumptions, the governing equation reduces to a linear second-order ordinary differential equation, and the total damping ($\delta_{\mathrm{tot}}$) is expressed as:

(1) $$\delta_{\mathrm{tot}}=\delta_{\mathrm{liq}}+\delta_{\mathrm{sh}}+\delta_{\mathrm{rad}}=\frac{4\mu_{L}}{\rho_{L}\omega_{0}R_{0}^{2}}+\frac{4\kappa_{s}}{\rho_{L}\omega_{0}R_{0}^{3}}+\frac{3\gamma P_{0}}{\rho_{L}\omega_{0}c_{L}R_{0}}$$

where ω_0_ is the bubble resonance angular frequency, $\kappa_{s}$ is shell viscosity coefficient ($\kappa_{s}=S_{f}/12\pi$), ρ_L_ is the density of the surrounding liquid, c_L_ is the speed of sound in the liquid, R is the bubble radius, and P_0_ is the hydrostatic pressure, γ is the polytropic coefficient (C_p_/C_v_, where C_p_ and C_v_ are the specific heat capacities of the gas at constant pressure and constant volume, respectively), and µ_L_ is the liquid viscosity [38], [42]. It has been shown that the largest contribution to the damping of 3 damping parameters in the case of a coated bubble is due to shell viscous damping [34], [39].

#### 2.1.1 Theory of acoustic attenuation in linear regime

Ultrasound energy is dissipated through scattering, as well as viscous, thermal, and shell damping. The combined effect is captured by the extinction cross section (σ_e_), which represents the total energy lost per bubble relative to the incident intensity [30], [44]. In the linear oscillation regime, under small amplitudes of oscillation with respect to the equilibrium radius ($R=R_{0}\left(1+\;\varepsilon \;\left(t\right)\right)$, with $\;\varepsilon \;\left(t\right)$ ≪ 1) the extinction cross section follows directly from the linearized bubble dynamics described above and is standardly expressed as [38]:

(2) $$\sigma_{e}=4\pi R_{0}^{2}\frac{c_{L}\delta_{\mathrm{tot}}}{\omega_{0}R_{0}}\frac{\Omega^{2}}{{\left(\Omega^{2}-1\right)}^{2}+\Omega^{2}\delta_{\mathrm{tot}}^{2}};\Omega =\frac{\omega_{0}}{\omega }$$

where ω is the angular driving frequency.

For a population of bubbles with number density n(a) and radius distribution a, the total attenuation $\alpha \left(\omega \right)$ in dB/cm is obtained by integrating σ_e_ over all bubble sizes (Eq. (3)). This model further assumes sufficiently low bubble concentrations, such that bubble–bubble interactions are negligible [30], [42].

(3) $$\alpha \left(\omega \right)=10\log_{10}e\int_{R_{\min}}^{R_{\max}}\sigma_{e}\left(a;\omega \right)n\left(a\right)da=10\left(\log_{10}e\right)n{(a)\sigma }_{e}$$

where R_min_ and R_max_ are the minimum and maximum bubble radii in the size distribution.

### 2.2 Nanobubble fabrication

NBs with distinct shell properties were formulated following protocols developed at Dr. Exner’s Laboratory at Case Western Reserve University [45]. Three NB formulations with controlled variation in shell composition were prepared as reported in [46]:

• PGG bubbles: lipid shell with added propylene glycol and glycerol
• PG bubbles: lipid shell with propylene glycol only
• G bubbles: lipid shell with glycerol only.

Propylene glycol acts as an edge activator and membrane softener, enhancing shell flexibility and enabling deformation without structural failure, thereby contributing to nonlinear oscillations. Glycerol narrows the size distribution and further stabilizes the bubbles by increasing the shell's elasticity, making it stiffer and potentially more prone to buckling under compression [47].

The lipid solution production process began by retrieving lipid vials from the freezer and allowing them to reach room temperature for 10 min. A water bath was prepared at 80°C, and a sonicator was set at room temperature (1510 Branson, Branson Ultrasonics Corp., CT, USA). To prepare a 10 mL vial of bubble solution, a 20 mL vial (Fisherbrand TM, PA, USA) was used with the following lipid amounts: 60.1 mg of DBPC (1,2-dibehenoyl-*sn*-*glycero*-3-phosphocholine) (Avanti ®, Sigma-Aldrich, MO, USA), 10 mg of DPPA (1,2-dipalmitoyl-*sn*-*glycero*-3-phosphate, sodium salt) (Avanti ®, Sigma- Aldrich, MO, USA), 20 mg of DPPE (1,2-Dipalmitoyl-*sn*-*glycero*-3-phosphoethanolamine) (CordenPharma, CO, USA), and 10 mg of DSPE-mPEG(2000) (1,2-distearoyl-n-*glycero*-3-phosphoethanolamine-N-[methoxy(polyethyleneglycol)2000] (ammonium salt) (Avanti ®,Sigma-Aldrich, MO, USA). Each lipid was measured on an electronic scale using weighing paper (Fisherbrand ®, PA, USA).

For PGG formulations, 1 mL propylene glycol was added to the lipid mixture, followed by incremental addition of a preheated solution containing 1 mL glycerol and 8 mL PBS. For PG formulations, 9 mL preheated PBS was added incrementally to lipids containing 1 mL propylene glycol, while for G formulations, 8 mL preheated PBS was added to lipids containing 2 mL glycerol. In all cases, additions were performed in 1 mL aliquots with gentle swirling and heating to minimize air entrainment. The resulting lipid suspensions were sonicated for 10 min at room temperature (Branson 1510) to ensure complete dissolution.

Following lipid dissolution and dispersion preparation in PBS, 1 mL aliquots were transferred to 3 mL vials. Air was removed and replaced with octafluoropropane gas (C_3_F_8_), followed by mechanical agitation (Bristol Myers Squibb, NY, USA) for 45 s. The resulting polydisperse suspensions were size isolated by differential centrifugation. To achieve a mean diameter of ∼650 nm, within the resonance range of 20 MHz transducers used in this experimental setup, the following protocol was developed:

Activated G, PG, and PGG bubble solutions were diluted in 12 mL of PBS in a 20 mL beaker and gently mixed. The diluted suspensions were transferred into 12 mL syringes (Becton Dickinson, NJ, USA), which were used as centrifugation vessels. To remove extra large bubbles, samples were sequentially centrifuged at 18g, 129g, and 135g for 2 min each, with the infranatant collected after each step, the foamy layer discarded, and the volume restored to 12 mL with PBS. To eliminate lipid debris and small NBs, samples were centrifuged at 200g for 2 min; the infranatant was discarded, and the foamy layer was resuspended in PBS. This washing step was repeated four times until the infranatant became optically clear, indicating removal of NBs smaller than ∼300 nm. The final foamy layer was concentrated in 3 mL PBS, transferred to a 20 mL scintillation vial, and used for subsequent experiments. Differential centrifugation was first introduced by Borden et al. [48]. The method was tailored for NB size control in this study.

### 2.3 Nanobubble size and concentration measurements

Size distribution and concentration of the size isolated NBs were measured using the Archimedes Particle Metrology System (Malvern Panalytical Instruments), which employs Resonant Mass Measurement (RMM) based on the Archimedes principle [49], [50]. The nanosensor used in this work detects buoyant and nonbuoyant particles ranging from 100 nm to 1.5 µm, enabling accurate differentiation between bubbles and lipid debris.

### 2.4 Experimental setup

**Fig. 1:**
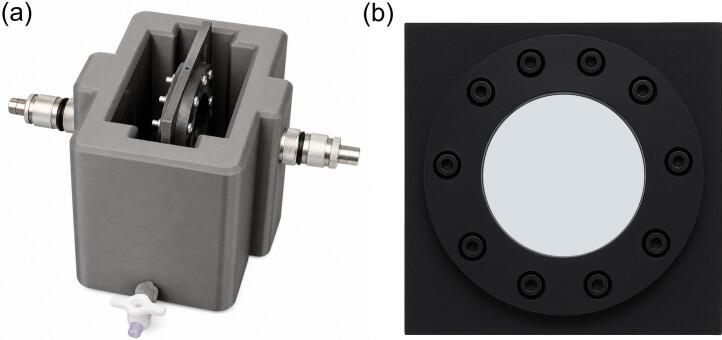
Custom-designed acoustic measurement hardware used for bulk attenuation experiments. (a) A 3D-printed water tank housing two coaxially aligned, unfocused 20 MHz flat immersion transducers operating in transmission–reception mode. (b) A 3D-printed sample chamber sealed with an acoustically transparent Mylar membrane. The chamber thickness was fixed at 5 mm to ensure the nanobubble suspension experiences a spatially uniform acoustic pressure field, minimizing artifacts from pressure gradients across the sample volume. This hardware configuration enables reproducible, broadband attenuation measurements of diluted nanobubble suspensions between 13–27 MHz.

**Fig. 2:**
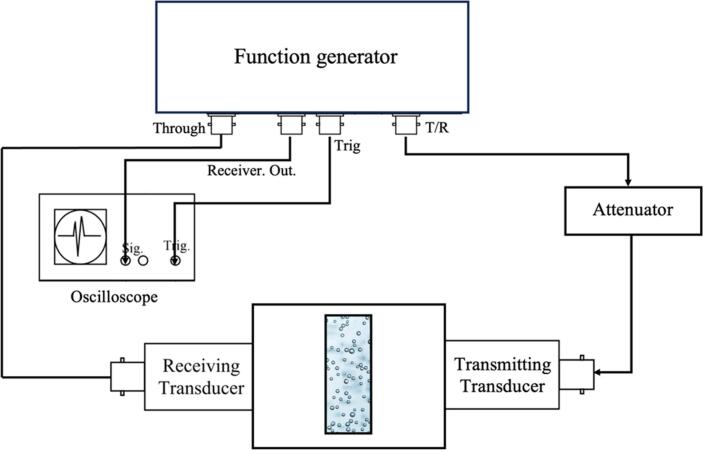
Full schematic of the broadband ultrasound attenuation measurement system. A short (∼500 ns) pulse drives the transmitting transducer, propagating an ultrasound wave through the sample chamber. The transmitted signal is captured by the receiving transducer and digitized by an oscilloscope. Attenuation spectra are computed by comparing the frequency-domain amplitude of the bubbly suspension signal against a water-only reference acquired under identical conditions. This transmission-based approach isolates the contribution of the nanobubbles to acoustic energy extinction across the full transducer bandwidth.

The ultrasound attenuation of diluted NB solutions was measured using a transmission–reception method similar to that in previous studies [29], [51]. A pair of unfocused flat immersion transducers (EVIDENT SCIENTIFIC, Webster, TX, USA) with a central frequency of 20 MHz, 9.52 mm element diameter, and 68% bandwidth (13.2–26.8 MHz) were mounted coaxially in a custom 3D-printed holder (Onyx micro-carbon fiber-reinforced nylon, Markforged, Massachusetts, USA) filled with water (see Fig. 1(a)). The sample chamber, also printed from Onyx, was placed in the acoustic path and sealed with an acoustically transparent Mylar membrane. To reduce the effect of non uniform driving pressure, the chamber width was limited to 5 mm, as shown in Fig. 1(b)[52]. A schematic of the whole setup is provided in Fig. 2.

The transmitting transducer was driven by a short (∼500 ns) pulse from a DPR300 Pulser–Receiver (Imaginant, Pittsford, USA). The resulting ultrasound wave propagated through the sample chamber and was attenuated by both the NBs and water. The receiving transducer detected the transmitted signal, which was then digitized using an oscilloscope (DSO-X 3024 series, Agilent Technologies, Santa Clara, CA, USA). A capsule hydrophone (ONDA HGL-series, ONDA Sunnyvale, CA, USA) was used to measure the axial pressure profile at different voltages, with a fixed distance of 3 cm from the transducer. This distance corresponds to the distance between the transducer and the sample holder used in our attenuation setup. Identical measurements were also performed on chambers containing only water to obtain reference signals. Each experiment was repeated n = 3 times (with new bubble activation from the same batch of lipids) for every shell type to confirm reproducibility.

Experimental attenuation curves were obtained at room temperature (∼25 °C) by comparing the Milli-Q water and bubbly water signals. Using Eq. (4), the measured attenuation data and NB number densities were incorporated into the theoretical model to estimate shell parameters across the frequency range of interest. The estimation procedure involved minimizing the sum of squared errors (E_r_) between the measured ($\alpha_{\exp}$) and modeled attenuation ($\alpha_{\mathrm{est}}$) curves. During parameter fitting, only shell stiffness (S_p_, varied between 0.1 and 10) and shell friction (S_f_, varied between 10^−9^–5 × 10^−8^) were adjusted to achieve the best agreement.

(4) $$E_{r}\left(S_{p},S_{f}\right)=\sum_{i}{\left[\alpha_{\mathrm{est}}{\left(f_{i}\right)}^{2}-\alpha_{\exp}{\left(f_{i}\right)}^{2}\right]}^{2}$$

### 2.5 Statistical analysis

To assess differences in shell stiffness and friction among the three NB formulations, we performed a two-way ANOVA. Reported p-values in the Results section indicate whether observed differences between groups were statistically significant.

## 3 Results

### 3.1 Size isolation and distribution

**Fig. 3:**
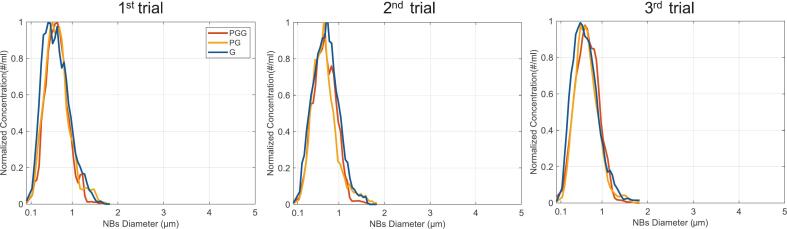
Size distributions confirm successful nanoscale size isolation across all three shell formulations. Normalized nanobubble (NB) concentration as a function of diameter, measured by Resonant Mass Measurement (Archimedes system, Malvern Panalytical) for three independent trials of PG, PGG, and G NBs. The Archimedes system differentiates buoyant NBs from nonbuoyant lipid debris based on buoyancy; over 92% of detected particles were buoyant in all trials, confirming suspension purity. All three formulations yield reproducible, narrow size distributions centered between 650–720 nm, a size range deliberately targeted to place the NB resonance frequency within the 13–27 MHz bandwidth of the measurement transducers. The consistency across trials validates the fabrication and differential centrifugation protocols as reliable routes to narrowly dispersed, shell-specific NB populations.

Following size isolation, the distribution of each bubble type was quantified using the Archimedes Particle Metrology System. A total of 1000 counts (buoyant and nonbuoyant particles) were acquired per trial, with over 92% corresponding to buoyant particles, confirming minimal presence of lipid aggregates and validating the efficiency of the isolation method. Fig. 3 shows the normalized NB concentration as a function of diameter for each trial, with the corresponding mean diameters (∼650–700 nm) and final concentrations (∼10^9^ NB/ml) summarized in Table 1.

**Table 1:** Mean diameter and concentration of three types of NBs for 3 trials measured with the Archimedes Particle Metrology System.

	Mean Diameter (nm)			Concentration ($\times 10^{9}NB/mL$)			Average Diameter (nm)	Average Concentration ($\times 10^{9}NB/mL$)
Shell Type	1st Trial	2nd Trial	3rd Trial	1st Trial	2nd Trial	3rd Trial		
PGG	698 ± 27	705 ± 26	740 ± 27	1.8	2.7	2.4	714 ± 28	2.3 ± 0.4
PG	654 ± 26	676 ± 27	650 ± 25	2.1	2.4	2.2	660 ± 13	2.3 ± 0.2
G	634 ± 24	700 ± 24	630 ± 23	2.2	3.1	2.4	655 ± 39	2.6 ±0.4

### 3.2 Acoustic attenuation measurements

**Fig. 4:**
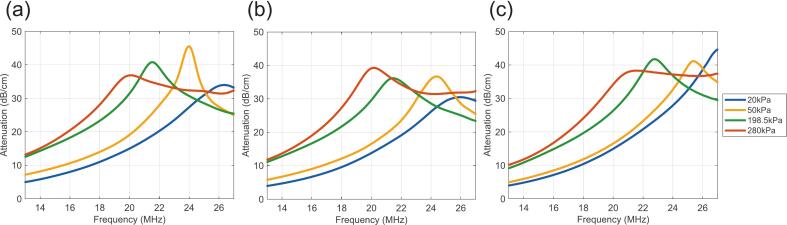
Attenuation spectra reveal shell-specific acoustic signatures and progressive nonlinear behavior with increasing pressure. Acoustic attenuation (dB/cm) versus frequency (MHz) for (a) PG, (b) PGG, and (c) G NBs at four representative driving pressures (20, 50, 198.5, and 280 kPa), shown for the first trial of each formulation. At 20 kPa (the lowest pressure tested, selected to ensure oscillations remain within the linear regime suitable for theoretical model fitting) the three formulations display distinct resonance frequencies that directly reflect their shell composition: G NBs resonate at the highest frequency (27.53 ± 0.21 MHz), followed by PGG (26.62 ± 0.26 MHz), and PG (25.84 ± 0.18 MHz), consistent with glycerol increasing shell stiffness and propylene glycol acting as a membrane softener that reduces it. At higher driving pressures (50, 198.5, and 280 kPa), the attenuation peak progressively broadens and shifts toward lower frequencies across all three formulations. By 280 kPa, the resonance frequency drops to 20.96 MHz (G), 20.26 MHz (PGG), and 19.51 MHz (PG), reflecting pressure-induced shell softening and the onset of nonlinear oscillation dynamics. These spectra form the primary experimental dataset from which resonance frequencies, spectral widths, and shell viscoelastic parameters are extracted throughout this study.

Attenuation measurements were performed on size-isolated PG, PGG, and G NBs across seven acoustic pressures (20, 35, 50, 138, 198, 241, and 280 kPa). Each experiment was repeated in triplicate to ensure reproducibility. Fig. 4 presents attenuation spectra for the first trial of each bubble type at four representative pressures (20, 50, 198, and 280 kPa). The concentrations used were $1.8\times 10^{8}$ NB/ml for PG NBs, $2.1\times 10^{8}$ NB/ml for PGG NBs, and $2.2\times 10^{8}$ NB/ml for G NBs. Full data for all trials and pressures are provided in Appendix A.

### 3.3 Resonance frequency

**Fig. 5:**
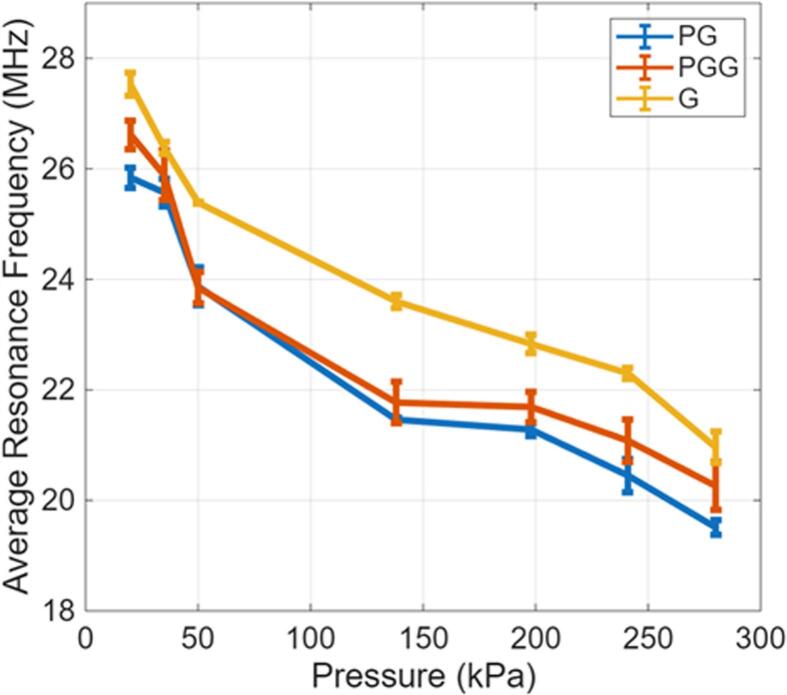
Shell composition governs both the magnitude and pressure sensitivity of nanobubble resonance frequency. Resonance frequency (MHz) as a function of acoustic driving pressure (kPa) for PG, PGG, and G NBs, with error bars representing standard deviation across three trials. At the lowest pressure (20 kPa), G NBs resonate near 27.5 MHz, PGG at an intermediate value, and PG near 25.8 MHz, directly reflecting the stiffening effect of glycerol and the softening effect of propylene glycol on the lipid shell. Across all formulations, the resonance frequency decreases monotonically with pressure by approximately 28% between 20 and 280 kPa, consistent with progressive shell softening and buckling under large-amplitude driving. PG and PGG NBs exhibit steeper frequency shifts than G NBs, confirming that more compliant shells are more susceptible to pressure-induced mechanical changes. Two-way ANOVA confirmed statistically significant effects of both pressure (p = 1.8 × 10^−11^) and shell type (p = 8.41 × 10^−7^).

For each sample, the resonance frequency was identified as the frequency at which maximum attenuation occurred. Fig. 5 shows the resonance frequencies for PG, PGG, and G NBs across all pressures, with error bars representing variability across three trials. Numerical values are provided in Appendix B. Peak attenuation values are also reported at each frequency in a table in Appendix C, followed by a figure showing how these peak values change across different acoustic pressures in Appendix D.

### 3.4 Shell parameter estimation

**Fig. 6:**
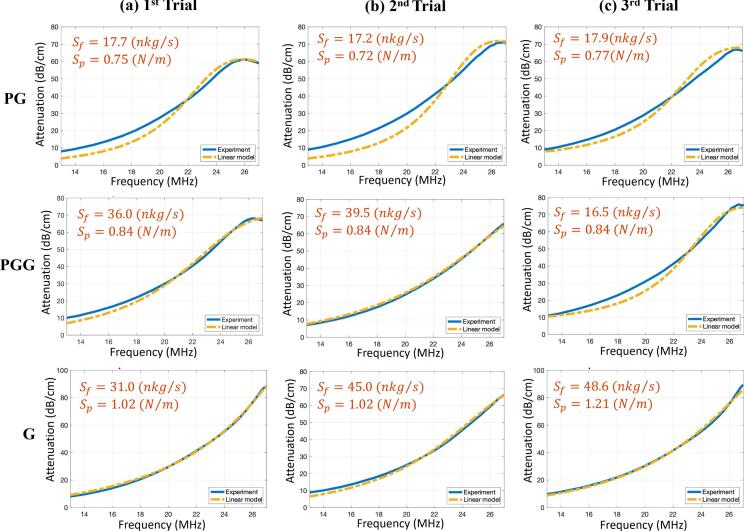
Theoretical model fits to experimental attenuation spectra enable shell parameter extraction in the linear regime. Experimental attenuation curves (solid lines) and best-fit theoretical predictions (dashed lines) at 20 kPa, the pressure confirmed to lie within the linear oscillation regime, for all three shell formulations (rows: PG, PGG, G) across three independent trials (columns a, b, c). Shell stiffness (S_p_) and friction (S_f_) were the only free parameters, optimized by minimizing the squared error between the model and the experiment, using experimentally measured size distributions as direct input. The estimated shell stiffness and shell friction values obtained from each fit are displayed in the corresponding panel and summarized across all trials in Table 2.

Shell parameters were determined at the lowest pressure (20 kPa) by fitting theoretical attenuation curves generated from the measured NB size distributions to the experimental attenuation data. Fig. 6 illustrates representative fits for each of the trials. Estimated S*_p_* and S*_f_* values are also listed in Table 2.

**Table 2:** Estimated shell stiffness ($S_{p}$) and shell friction ($S_{f}$) of three types of NBs for 3 trials using the linear bubble oscillation model.

Parameter	NB type	1st Trial	2nd Trial	3rd Trial	Average
$S_{f}\times 10^{-8}\left(\frac{kg}{s}\right)$	**PG**	1.77	1.72	1.79	**1.76**±**0.03**
	**PGG**	3.60	3.95	1.65	**3.06**±**1.23**
	**G**	3.10	4.50	4.86	**4.08**±**1.04**
$S_{p}\left(\frac{N}{m}\right)$	**PG**	0.75	0.72	0.77	**0.75**±**0.02**
	**PGG**	0.84	0.84	0.84	**0.84**±**0.00**^1^
	**G**	1.02	1.02	1.21	**1.08**±**0.10**

^1^Standard deviation is zero within numerical precision; uncertainty from measurement noise is not captured.

## 4 Discussion

### 4.1 Pressure-dependent resonance frequency

**Fig. 7:**
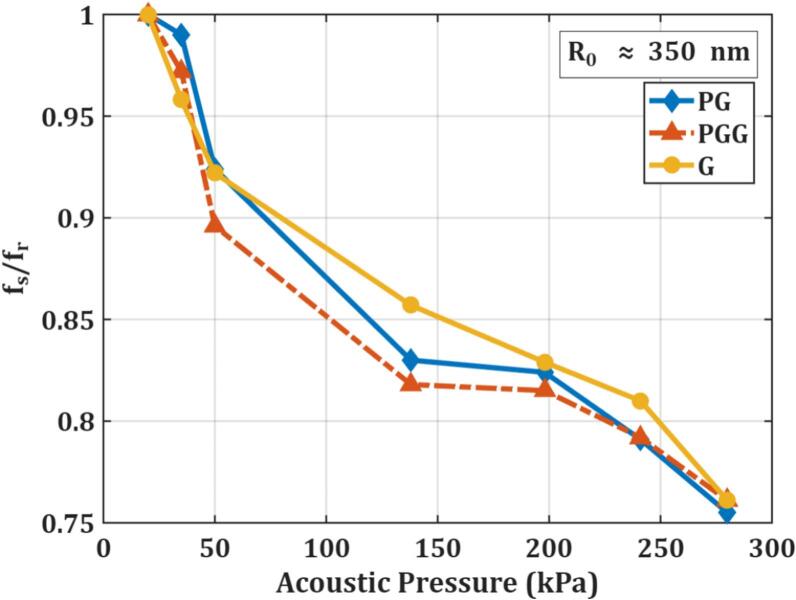
Normalized resonance frequency shift isolates pressure-induced shell softening independently of bubble size. The resonance frequency at each driving pressure (f_s_) normalized by the linear-regime resonance frequency at 20 kPa (f_r_), plotted as a function of acoustic pressure for all three NB formulations. Normalizing by f_r_ removes the influence of absolute bubble size, allowing the purely pressure-driven mechanical response of each shell type to be compared directly. All formulations show a monotonic decline in f_s_/f_r_, confirming progressive shell softening with increasing pressure. PG and PGG NBs display steeper normalized shifts than G NBs, establishing that shell composition (not bubble size) is the dominant factor controlling how rapidly the resonance frequency drops with pressure. This representation also facilitates direct comparison with prior microbubble studies that report similar normalized shifts, situating the present NB findings within the broader bubble mechanics literature.

A consistent downshift in resonance frequency with increasing acoustic pressure was observed for all shell types. This trend aligns with theoretical predictions from models such as Marmottant’s, which describe transitions from elastic to large amplitude oscillations in which shell elasticity diminishes due to shell buckling [30], [53]. Prior studies on MBs reported similar behavior, with lipid shells softening under high pressure and lowering the resonance frequency [34], [51], [54], [55]. Fig. 7 presents the shift of the resonance frequency at higher pressures (*f_s_*) normalized by the linear resonance frequency (*f_r_*).

Statistical analysis confirmed that both acoustic pressure (p-value = 1.8 × 10^−11^) and shell type (p-value = 8.41 × 10^−7^) significantly influenced resonance frequency. Notably, PG and PGG bubbles exhibited greater pressure sensitivity than G bubbles. These findings establish that for bubble populations of comparable size, resonance frequency shifts are primarily dictated by shell composition and excitation pressure.

### 4.2 Width of the attenuation curves

**Fig. 8:**
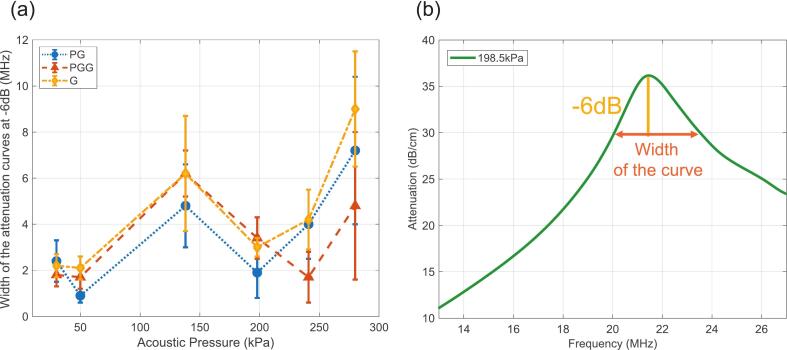
Non-monotonic spectral bandwidth reveals distinct pressure-dependent dynamical regimes in nanobubble oscillation. (a) The −6 dB width of the attenuation spectrum (MHz), a measure of oscillation bandwidth, plotted versus acoustic driving pressure for PG, PGG, and G NBs. Error bars show standard deviation across three trials; the 20 kPa point is excluded due to transducer bandwidth limitations. Rather than increasing monotonically with pressure, the bandwidth follows a non-monotonic pattern that identifies four dynamical regimes: (i) quasi-linear oscillations below 50 kPa (narrow bandwidth, ∼2 MHz); (ii) a transition zone near 140 kPa where bandwidth abruptly widens to 6–8 MHz as heterogeneous shell responses emerge across the population; (iii) a narrowing near 200 kPa hypothesized to reflect a stable, periodic buckling–unbuckling regime, particularly pronounced in the more compliant PG shell; and (iv) a high-variability zone above 250 kPa consistent with shell rupture, lipid shedding, or bubble fragmentation. (b) Schematic illustrating how the − 6 dB bandwidth is extracted from a representative attenuation spectrum, defined as the frequency span over which attenuation remains within 6 dB of its peak value.

The experimental attenuation spectra provide a window into the dynamics of NBs, highlighting fundamental shell behaviors. Among the extracted features, the variation in peak width with increasing acoustic pressure (as shown in Fig. 4) is noteworthy. At −6 dB attenuation, peak width initially increases for all shell types up to 138 kPa, then decreases until ∼198 kPa, after which it increases again (Fig. 8). The 20 kPa case was excluded due to transducer bandwidth limitations.

This non-monotonic behavior likely reflects pressure-dependent transitions in shell mechanics, with specific thresholds at which different NB subpopulations exhibit enhanced attenuation or altered oscillation modes. The data suggest four distinct regimes for the range of pressures examined: (a) Quasi-linear zone (<50 kPa): All curves exhibit narrow bandwidths (∼2 MHz), consistent with small amplitude, approximately linear oscillations. (b) Transition zone (∼140 kPa): The bandwidth abruptly increases to 6–8 MHz, indicating the onset of nonlinear responses and heterogeneous shell behavior across the population. (c) Buckling zone (∼200 kPa): The bandwidth narrows again (to ∼2 MHz for PG NBs). We hypothesize that this dip corresponds to a stable buckling regime in which the compliant PG shell “snaps” into a buckled configuration, producing highly periodic, organized oscillations dominated by repetitive buckling–unbuckling cycles. (d) Destruction zone (>250 kPa): Increased bandwidth variability and elevated standard deviations suggest shell rupture, lipid shedding, or other disruptive events. Under these pressures, strong periodic driving disrupts bubble integrity, leading to broadband, noise-like spectra as populations fragment or collapse.

Although a complete mechanistic explanation is beyond the scope of this study, these observations underscore the complexity of pressure-dependent shell dynamics and highlight the need for future work to map these nonlinear regimes more precisely.

### 4.3 Shell parameter estimation method

**Fig. 9:**
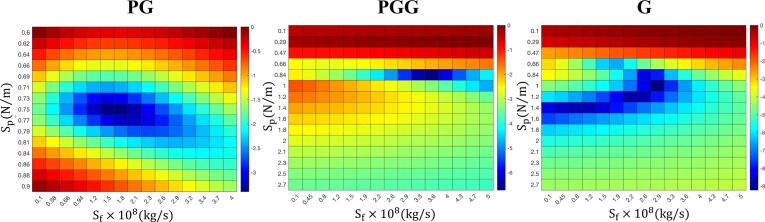
Well-defined minima in the error landscape confirm unique and reproducible shell parameter identification. Two-dimensional heat maps of the logarithmic fitting error (Eq. (4), normalized by 1 (dB/cm)^4^) plotted over the shell stiffness (S_p_, N/m) and shell friction (S_f_, kg/s) parameter space, shown for the first trial of PG (left), PGG (center), and G (right) NBs. Each map was generated by evaluating the mismatch between theoretical and experimental attenuation curves over a dense parameter grid. The presence of a single, well-isolated minimum in each panel confirms that the inverse problem is identifiable, i.e., a unique combination of S_p_ and S_f_ best explains the measured attenuation, and rules out significant parameter degeneracy. The minimum shifts systematically across formulations for shell stiffness: PG occupies the lowest S_p_, followed by PGG, with G exhibiting the highest value, directly reflecting how propylene glycol softens and glycerol stiffens the lipid shell. For shell friction, PG again shows the lowest S_f_ value, while PGG and G display comparable friction in this trial, suggesting that G and the combined PGG formulation produce similarly dissipative shells despite their differences in stiffness.

Shell parameters were estimated in the linear regime (20 kPa) to minimize nonlinear effects. By fitting theoretical attenuation curves to experimental spectra using NB size distributions from RMM measurements, optimal values for S*_p_* and S*_f_* were obtained. Error function minimization across parameter grids (Fig. 9) revealed the model's sensitivity, with well-defined minima identifying the best-fit values.

### 4.4 Comparison between estimated shell parameters of PGG, PG, and G NBs

A one-way ANOVA with Bonferroni correction demonstrated statistically significant differences in S*_p_* and S*_f_* among the three NB types (p = 0.0018 for S*_p_*, and p = 0.0459 for S*_f_*). PG NBs showed the lowest stiffness, consistent with the membrane softening role of propylene glycol, whereas G NBs exhibited the highest stiffness and friction due to glycerol’s stiffening effect. PGG NBs displayed intermediate values, with their frictional properties resembling G NBs, suggesting that glycerol’s influence dominates when combined with propylene glycol. Post-hoc pairwise comparisons were also performed to identify the shell groups with the greatest differences in shell parameters. The resulting comparisons for S*_p_* and S*_f_* are summarized in Table 3.

**Table 3:** The post hoc analysis with the Bonferroni correction for the *S_p_* and *S_f_* values among 3 different groups of PG, PGG, and G NBs. The test statistic (p-value ≤ 0.0167) indicates that PG and G NBs have different shell stiffness values.

Shell Parameter	Groups	p-value (T-test)
$S_{p}$	**PG vs PGG**	**0.0030**
	**PG vs G**	**0.0066**
	PGG vs G	0.0184
$S_{f}$	PG vs PGG	0.1420
	**PG vs G**	0.0112
	PGG vs G	0.2912

### 4.5 NBs vs MBs shell parameters

To the best of the authors’ knowledge, no published studies have reported estimates of shell elastic parameters for lipid-coated bubbles in the submicron range, making the present work the first of its kind. Comparisons across studies are further complicated by the fact that bubble size, shell composition, and gas core vary widely in the literature. The closest direct comparison available is the study by Rostam Shirazi et al. [35], which used the same shell formulation and gas core but examined considerably larger bubbles (mean diameter ≈ 3.4 μm). In that study, both *S_p_* and *S_f_* are substantially lower in the present NBs. For example, the shell friction of G bubbles decreases from 197 × 10^−8^ kg/s in MBs to 4.08 × 10^−8^ kg/s in NBs.

The smallest bubbles previously analyzed for shell parameter estimation are those reported by Helfield et al. [33], with a diameter of 1.02 μm (Table 4). However, that study used a different shell composition (DSPC-based) and a C_4_F_10_ gas core. The estimation method, bulk acoustic attenuation fitted to a linear oscillation model, was the same as in the present work, and the experimental resonance frequency was 19.5 MHz. Their reported parameters were *S_p_* = 1.76 N/m and *S_f_* = 0.08 × 10^−^⁶ kg/s. When comparing these values with those obtained here, *S_p_* appears less sensitive to bubble size and/or frequency, decreasing from 1.76 N/m (MB, R_0_ = 1.02 μm) to 0.84 N/m (PGG NBs), a 52% reduction. In contrast, *S_f_* decreases more sharply, from 8 × 10^−8^ kg/s (MBs) to 3.06 × 10^−8^ kg/s (PGG NBs), corresponding to a 67% reduction.

**Table 4:** A summary of the NB estimated shell parameters obtained in the present work, as well as shell parameters and bubble properties from a similar study: Helfield et al. [33] with different bubble formulation.

References	Bubble type	Frequency (MHz)	Average size (μ m)	$S_{p}\left(\frac{N}{m}\right)$	$S_{f}\times 10^{-8}\left(\frac{kg}{s}\right)$
Present study	PG NBs	13–27	0.67 ± 0.01	0.75 ± 0.02	1.76 ± 0.03
	PGG NBs	13–27	0.72 ± 0.03	0.84 ± 0.00	3.06 ± 1.23
	G NBs	13–27	0.65 ± 0.04	1.08 ± 0.10	4.08 ± 1.04
Helfield et al.	MBs	4–16	1.02 ± 0.02	1.76 ± 0.15	8 ± 2

Previous single-bubble investigations have also shown that shell friction decreases as bubble size decreases [17], [18]. Those studies attributed this trend to the higher interfacial shear rates experienced by smaller bubbles and to the shear-thinning behaviour characteristic of phospholipid monolayers. Our findings suggest that this behaviour extends into the nanoscale regime (∼600 nm diameter) and persists at excitation frequencies above 20 MHz. This has important implications for high-frequency contrast imaging: because shell-induced damping ($\delta_{\mathrm{sh}}$) exhibits an inverse cubic dependence on radius (Eq. (1)), smaller bubbles would normally be expected to experience reduced oscillation amplitudes. A size-dependent reduction in *S_f_* counteracts this effect, allowing NBs to sustain larger oscillation amplitudes and potentially stronger nonlinear responses. It should also be emphasized that existing experimental approaches cannot fully separate the effects of bubble size from those of driving frequency when extracting shell parameters. As demonstrated by Helfield et al. (2014), even for a fixed MB population, the fitted stiffness and friction vary with the ultrasound frequency used for measurement [33]. As the resonance frequency depends on $R_{0}$, changing the bubble size inevitably shifts the frequency range at which attenuation is evaluated. Consequently, apparent trends in shell stiffness or friction with bubble size may partially reflect differences in the measurement frequency rather than intrinsic size-dependent mechanics.

The unexpectedly lower *S_p_* measured for NBs compared with larger lipid-coated MBs (of different formulations) may reflect intrinsic nanoscale effects that are not captured by conventional MB scaling. As bubble radius decreases, surface curvature increases markedly (∝1/R), which can give rise to several curvature-driven mechanisms. One possible explanation is that the high curvature of NBs leads to tighter lipid packing and increased local surface charge density, thereby altering electrostatic interactions within the lipid monolayer and softening the effective shell mechanics; similar charge-mediated effects have been reported in MBs when varying the salinity or PBS concentration of the surrounding medium [56]. In addition, the very high curvature of a 600 nm diameter interface alters lipid head group spacing, chain tilt, and packing order. Such curvature-induced structural changes are known to reduce the effective dilatational modulus under dynamic excitation, as demonstrated by molecular and coarse-grained simulations, as well as membrane mechanics studies [57]. Curvature can also influence the behaviour of PEGylated lipids: PEG-lipid chains can shift between “mushroom” and “brush” regimes depending on local curvature and grafting density, which alters lateral pressure and therefore the apparent shell stiffness [58]. Experimental studies [58] show that PEG fraction strongly affects measured surface moduli, indicating that the PEG-containing formulation used here may be particularly sensitive to nanoscale curvature effects. Finally, Marmottant-type lipid shells exhibit buckling, rupture, and nonlinear surface tension behaviour, and both the buckling pressure and the small strain effective modulus depend on curvature-induced prestress (i.e., Laplace pressure). If 600 nm NBs operate closer to partially buckled or prestressed states, the fitted linear S_p_ would naturally appear lower. Shell model studies emphasize that these nonlinearities can significantly influence parameter extraction and interpretation [59].

Overall, comparisons with prior MB studies show that both the fitted *S_p_* and the fitted *S_f_* tend to decrease as the bubble radius decreases; several experimental and modelling studies report similar size trends and interpret them in terms of interfacial rheology that is rate-dependent rather than strictly Newtonian. In particular, theoretical models and single-bubble analyses indicate that shell friction decreases as the interfacial shear rate increases (i.e., shear-thinning behaviour) [60]. Experimental work on phospholipid-coated MBs has also reported a decrease in fitted viscous parameters with decreasing bubble size and with increased excitation conditions, consistent with shear-thinning interpretations [61]. The evidence for shear-thinning in our NBs is suggestive but not definitive: our bulk attenuation measurements sample an ensemble response and are sensitive to polydispersity, bubble–bubble interactions, and model identifiability, any of which can bias fitted parameters [33], [62]. Therefore, while our results are consistent with extending shear-thinning rheology to the nanoscale, alternative explanations (curvature-dependent packing, PEG conformational changes, Laplace prestress, or ensemble effects) remain plausible and require further targeted experiments.

### 4.6 Effect of size isolation process on shell formulation

Differences in preparation methods may also contribute to the observed shell parameter values. Our use of differential centrifugation with multiple foam dilutions in PBS could alter shell formulation and thus affect the lipid packing compared to previous MB studies [35], [63]. Such methodological variations complicate direct comparisons of shell parameters between NBs and MBs. A systematic investigation isolating preparation effects from size related effects is therefore warranted.

### 4.7 Key Novel Contributions of This Work

This study demonstrates, for the first time, that the elastic shell parameters of lipid-coated NBs can be quantified experimentally using bulk acoustic attenuation measurements. By integrating a highly stable NB formulation with precise size isolation methods and rigorous statistical fitting, we show that both shell composition and excitation pressure significantly modulate NB acoustic behaviour. These results extend shell mechanics characterization into the submicron regime, an area previously inaccessible, and help bridge the long-standing gap between NB and MB modelling. This establishes a foundation for rationally tuning NB shell formulations for high-frequency imaging and ultrasound-mediated drug delivery.

A second major contribution of the present work is the use of a bulk attenuation approach that captures the ensemble response of NB populations that more closely mimics clinical intravenous administration. Unlike single-bubble interrogation methods, bulk measurements reflect the average mechanical behaviour of thousands of bubbles, making them particularly valuable when bubble-to-bubble variability is significant. Recent single-bubble studies, such as the work by Spiekhout et al. (2024) [62] on ∼ 2000 microfluidically generated MBs (R_0_ ≈ 2.4 μm), illustrate this variability clearly. Even with identical shell formulations, they reported a wide range in shell friction (from 2 × 10^−9^ to 2 × 10^−8^ kg/s) and resonance frequencies that varied by a factor of 2 (1.7–3.5 MHz). Such findings highlight that nominally identical bubbles can differ substantially in their elastic properties, underscoring the value of ensemble based methods, such as bulk attenuation, for capturing clinically relevant behaviour.

## 5 Conclusion and future work

We produced three phospholipid-coated NB formulations (PG, PGG, and G) with distinct viscoelastic behaviour, and isolated size distributions (mean diameters ≈ 650–700 nm) at concentrations of order 10^9^ NB/ml. At low pressure (20 kPa), the measured resonance frequencies depended on shell composition (PG ≈ 25.8 MHz, PGG intermediate, G ≈ 27.5 MHz), and increasing the acoustic pressure to 280 kPa produced a consistent downward shift in resonance (∼28%), reflecting pressure sensitive shell mechanics. Model fitting of the attenuation spectra indicates that G bubbles are the stiffest and most dissipative, while PG bubbles are the most compliant. These differences are statistically significant (ANOVA with Bonferroni correction for pairwise comparisons).

Overall, this work establishes that lipid shell composition significantly influences NB resonance and viscoelastic response and provides a reproducible bulk measurement framework for comparing formulations. However, because the inference of underlying rheology from ensemble attenuation is indirect, future single-bubble or interfacial rheology measurements are needed to confirm shear-thinning and to decouple size, frequency, and pressure effects more fully.

We note that the NBs studied here (≈650–700 nm) are larger than those employed in many emerging NB applications, where reported diameters often fall below 300 nm. As bubble size decreases further, increased curvature and Laplace pressure are expected to amplify curvature-driven effects on lipid packing, surface charge density, and shell prestress, potentially leading to even softer and more nonlinear effective shell mechanics. Extrapolating the present trends to smaller NBs therefore suggests that size-dependent deviations from conventional microbubble scaling may become increasingly pronounced.

While our present study provides valuable insights into NB shell elasticity and friction under relatively low pressure conditions, it is limited in fully capturing nonlinear shell dynamics that may emerge at clinically relevant higher pressures. To address this, future investigations will extend into the nonlinear oscillation regime by measuring attenuation curves over a broad pressure range (50–280 kPa). We propose a two-step modelling strategy that combines a modified Rayleigh–Plesset equation (to describe radial bubble motion under strong driving) with a Caflisch derived effective medium equation (to account for wave propagation and ensemble interactions in a suspension) [64], [65]. This combined approach will help us disentangle how shell stiffness and friction evolve with large amplitude deformations and collective effects, thus providing deeper insight into pressure-dependent shell mechanics, a regime underexplored in current studies (e.g., nonlinear analyses of microbubble propagation highlight the role of buckling, rupture, and shell viscoelasticity in attenuation and dispersion) [66].

Another promising research direction is to systematically investigate the influence of surface charge (e.g., zeta potential) on NB shell behaviour. Changes in ionic strength or charged lipid composition have been shown to alter MB shell properties significantly: for instance, increasing salinity causes a notable drop in both stiffness and shell friction, likely via charge layer formation and altered interfacial tension [67], [56]. By measuring zeta potential alongside acoustic attenuation and performing shell parameters fitting under varying ionic conditions, we can clarify how electrostatic interactions modulate mechanical properties. This will directly address a key limitation of our current work, which assumes that shell mechanics are independent of the ionic environment, and could lead to optimized formulations tailored for different physiological conditions (e.g., blood, saline, or drug loaded environments).

## CRediT authorship contribution statement

**Mahshid Yaali:** Writing – review & editing, Writing – original draft, Visualization, Validation, Supervision, Methodology, Investigation, Formal analysis, Data curation, Conceptualization. **Agata A. Exner:** Writing – review & editing, Supervision, Project administration, Investigation, Funding acquisition. **Michael C. Kolios:** Writing – review & editing, Validation, Supervision, Resources, Project administration, Investigation, Funding acquisition.

## Declaration of competing interest

The authors declare the following financial interests/personal relationships which may be considered as potential competing interests: Agata A. Exner is a founder of Visano Theranostics, a company that develops nanobubble-based technologies. The research presented in this manuscript was conducted independently and was not influenced by the company. The remaining authors declare that they have no known competing financial interests or personal relationships that could have appeared to influence the work reported in this paper.

## Figures and Tables

**Fig. A:**
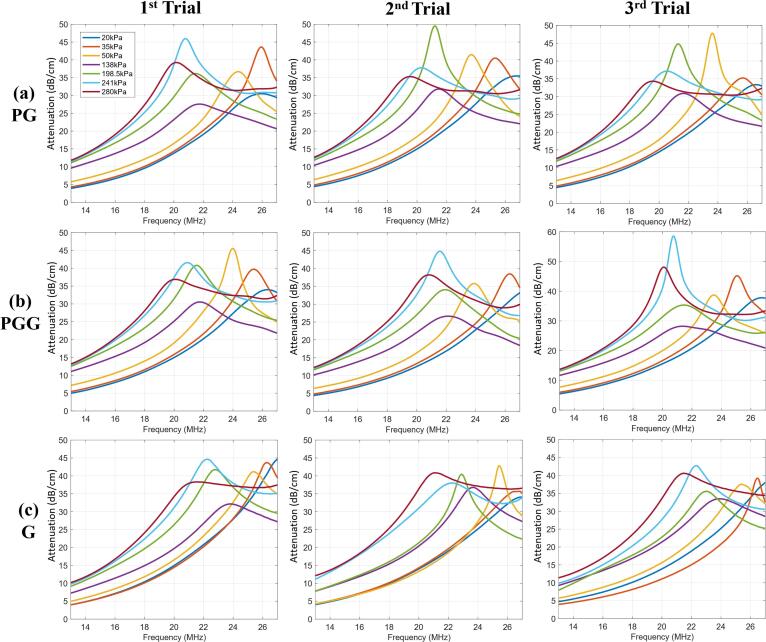
Complete pressure-dependent attenuation spectra for all shell formulations and trials. Attenuation (dB/cm) versus frequency (MHz) for (a) PG, (b) PGG, and (c) G NBs across all seven driving pressures (20–280 kPa) and all three independent trials. These data are the complete experimental record underlying the resonance frequency, spectral width, and their pressure dependency presented in the main text. The consistent pattern of resonance downshift and peak broadening with increasing pressure, reproduced across all trials and formulations, confirms that the observed pressure-dependent behavior is a robust property of each shell type rather than a trial-specific artifact.

**Table B:** Pressure-dependent resonance frequencies for all three nanobubble formulations. Mean resonance frequency (MHz) ± standard deviation across three independent trials for PG, PGG, and G NBs at each of the seven acoustic driving pressures tested (20–280 kPa). The resonance frequency was identified as the frequency of peak attenuation in each measured spectrum, extracted directly from the attenuation curves presented in Appendix A. Across all formulations, resonance frequency decreases monotonically with increasing pressure, with G NBs consistently resonating at the highest frequency and PG at the lowest at every pressure level, reflecting the relative stiffness imparted by their respective shell compositions.

		Average Resonance Frequency (MHz)	
Pressure (kPa)	PG	PGG	G
20	25.84 ± 0.18	26.62 ± 0.26	27.53 ± 0.21
35	25.57 ± 0.25	25.88 ± 0.45	26.38 ± 0.11
50	23.87 ± 0.34	23.85 ± 0.29	25.39 ± 0.02
138	21.46 ± 0.04	21.77 ± 0.37	23.60 ± 0.12
198	21.28 ± 0.11	21.69 ± 0.27	22.83 ± 0.17
241	20.45 ± 0.30	21.08 ± 0.38	22.30 ± 0.10
280	19.51 ± 0.14	20.26 ± 0.44	20.96 ± 0.28

**Table C:** Peak attenuation at the pressure-dependent resonance frequency for all three nanobubble formulations. Mean maximum attenuation (dB/cm) for PG, PGG, and G NBs, measured at the resonance frequency corresponding to each driving pressure, extracted from the attenuation spectra presented in Appendix A. Rather than increasing monotonically with pressure, peak attenuation values fluctuate across the pressure range for all three formulations, reflecting the combined influence of pressure-induced resonance frequency shifts, which move the attenuation peak relative to the transducer bandwidth, progressive shell softening, and the onset of nonlinear oscillation modes at higher driving pressures. These values are plotted graphically in Appendix D.

		Average Maximum attenuation (dB/cm)	
Average Resonance Frequency (MHz)	PG	PGG	G
25.84	33.07	35.53	34.06
25.57	39.76	41.12	39.51
23.87	41.96	39.99	40.44
21.46	30.18	28.43	34.12
21.28	43.33	36.71	39.22
20.45	40.27	48.28	41.76
19.51	36.23	41.00	39.79

**Fig. D:**
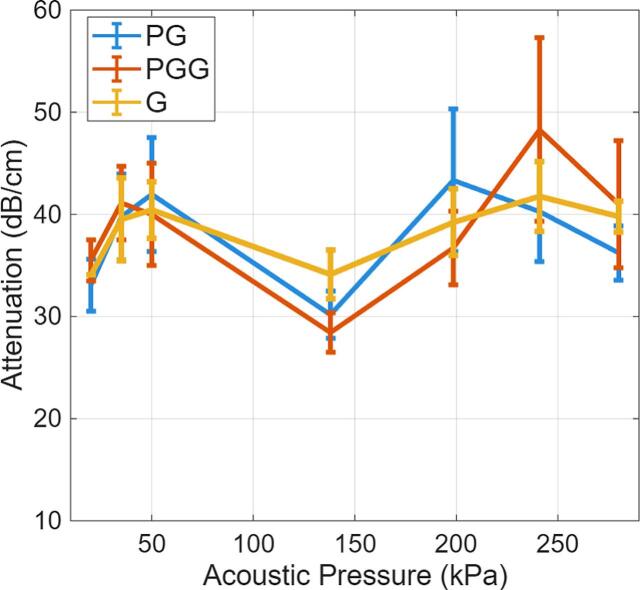
Peak attenuation amplitude varies non-monotonically with driving pressure, reflecting competing shell dynamics. Mean peak attenuation (dB/cm) versus acoustic driving pressure for PG, PGG, and G NBs, plotted from the numerical values tabulated in Appendix C. All three formulations display non-monotonic behavior, reflecting the interplay between pressure-induced resonance frequency shifts, progressive shell softening, and the onset of nonlinear and destructive oscillation modes at higher pressures.
